# Bimanual coupling paradigm as an effective tool to investigate productive behaviors in motor and body awareness impairments

**DOI:** 10.3389/fnhum.2013.00737

**Published:** 2013-11-05

**Authors:** Francesca Garbarini, Lorenzo Pia

**Affiliations:** ^1^Department of Psychology, University of TurinTurin, Italy; ^2^Neuroscience Institute of Turin, University of TurinTurin, Italy

**Keywords:** productive behaviors, bimanual coupling, anosognosia for hemiplegia, delusion of ownership, somatoparaphrenia, body awareness, motor awareness, right brain-damaged patients

## Abstract

When humans move simultaneously both hands strong coupling effects arise and neither of the two hands is able to perform independent actions. It has been suggested that such motor constraints are tightly linked to action representation rather than to movement execution. Hence, bimanual tasks can represent an ideal experimental tool to investigate internal motor representations in those neurological conditions in which the movement of one hand is impaired. Indeed, any effect on the “moving” (healthy) hand would be caused by the constraints imposed by the ongoing motor program of the ‘impaired’ hand. Here, we review recent studies that successfully utilized the above-mentioned paradigms to investigate some types of productive motor behaviors in stroke patients. Specifically, bimanual tasks have been employed in left hemiplegic patients who report illusory movements of their contralesional limbs (anosognosia for hemiplegia). They have also been administered to patients affected by a specific monothematic delusion of body ownership, namely the belief that another person’s arm and his/her voluntary action belong to them. In summary, the reviewed studies show that bimanual tasks are a simple and valuable experimental method apt to reveal information about the motor programs of a paralyzed limb. Therefore, it can be used to objectively examine the cognitive processes underpinning motor programming in patients with different delusions of motor behavior. Additionally, it also sheds light on the mechanisms subserving bimanual coordination in the intact brain suggesting that action representation might be sufficient to produce these effects.

Studying the neurocognitive mechanisms underpinning bimanual movements has a key role for the knowledge of motor cognition. Indeed, in daily life, most actions are complex and require the integration of bimanual movements. As an attempt to achieve a better understanding of complex movements in ecological conditions, scientists introduced at the end of the 70’s bimanual tasks in addition to reaction time during simple key-pressing tasks, mostly employed in the classical motor-control literature. The new methodology evidenced fundamental constraints subserving bimanual actions, as strong coupling effects arise and neither hand is able to perform simultaneous independent actions. A crucial point that emerges from the literature is that such limitations are strongly linked to central operations (i.e., internal representation of action), rather than to online information arising from the periphery (i.e., action execution). Hence, it is possible to hypothesize that these constraints would be also present in pathological conditions in which movements are impaired as a consequence of brain damage.

Studies with brain-damaged patients affected by action disorders provide a unique opportunity to test the above-mentioned hypothesis. Here, we briefly review some studies employing bimanual coupling paradigms to investigate productive behaviors in neurological patients affected by motor or body awareness deficits. In the motor domain, we will focus on patients affected by anosognosia for hemiplegia (AHP) who, despite the presence of severe paralysis, obstinately claim that they can still move their paralyzed limb. In the body domain, we will examine patients with an unusual form of contralesional asomatognosia, who claim that the examiner’s hand is their own whenever it is positioned, in egocentric coordinates, next to their own left hand.

## BIMANUAL COUPLING PARADIGMS

When people move both hands at the same time, strong coupling effects arise, such that the two hands are constrained in spatial or temporal terms (see Swinnen, 2002; Franz, 2003 for reviews). The temporal and spatial interferences, known as bimanual coupling, seem to be dissociable both functionally and anatomically (e.g., Heuer, 1993; Franz et al., 1996). In the spatial domain, coupling can pertain both to movement amplitude and direction (see Swinnen et al., 2003; Wenderoth et al., 2004 for reviews). Amplitude parameters have been manipulated by having subjects to perform left and right limb movements with same or different amplitude specifications (see Swinnen, 2002; Swinnen et al., 2002). In the spatial directional domain, perhaps the most employed paradigm to reveal the reciprocal influence of hand actions requires people to simultaneously draw lines with one hand and circles with the other. Here, coupling consists in the fact that participants tend to produce curved lines and line-like circles (Franz et al., 1991). It is worth noticing that similar constraints can also be observed in drawing tasks involving more discontinuous shapes, such as squares combined with circles (Franz, 2003).

As regards to the temporal domain, whereas in unimanual reaching movements exists a reliable temporal relationship between difficulty and time, when the same movements are combined in a bimanual task where the difficulty of the is varied (target distance, width, or both), the two hands initiate and terminate in a more coupled fashion (Kelso et al., 1979). Similarly, when people are asked to tap rhythms with both two fingers using non-harmonic relations, they are unable to produce two clearly distinct timing patterns without any interference (Peters, 1977). Nonetheless, bimanual coordination in the mirror-symmetrical (in-phase) mode (i.e., homologous muscles are active simultaneously) is more stable than in the anti-parallel (out-of-phase) mode [i.e., homologous muscles are engaged in an alternating fashion; see (Swinnen et al., 2002)]. When subjects bimanually rotate disks with their index fingers in the out-of-phase mode, for example, increasing the movement frequency ultimately results in a transition towards the in-phase mode, but the opposite transition does not occur (Kelso, 1984).

From an anatomical point of view, converging neuroimaging evidence shows that, in right-handed subjects, the (left) dominant hemisphere plays a crucial role in performing bimanual symmetrical (in phase) movements, whereas the (right) non-dominant hemisphere has a key role during the execution of bimanual asymmetrical (out of phase) movements. Within the asymmetric movements network, increased activity has been observed in the dorsal premotor cortex, the cingulate motor area (CMA), the supplementary motor area (SMA), the posterior parietal cortex (PPC), and the cerebellum (Meyer-Lindenberg et al., 2002; Wenderoth et al., 2004; Aramaki et al., 2006; Maki et al., 2008). A very recent neuroimaging study (Garbarini et al., in press), employing both executed and imagined tasks, found two different components of bimanual coupling: one strictly related to the execution of the bimanual movement, the other to the abstract selection of the (non-congruent) motor programs. This last component was found to be equal for both motor execution and motor imagery. Specifically, a prefrontal-parietal network (mostly involving right pre-SMA/CMA and bilateral PPC) subserves bimanual coupling effects independently form the condition (execution or imagery).

The presence of a unique brain network underpinning coupling in the execution and imagination of bimanual movements is consistent with the idea that supraspinal mechanisms (e.g., motor programs, sensory predictions, abstract representation of actions) might be sufficient to produce these effects. Indeed, behavioral studies showed coupling effects even in absence of somatosensory consequences (e.g., patients with peripheral sensory loss Drewing et al., 2004; Spencer et al., 2005 or visual feedback Cardoso de Oliveira and Barthelemy, 2005; Spencer et al., 2005). Besides, further studies showed that the interferences could not be modulated manipulating afferent sources of information, confirming that the spatial interference primarily emerges at the level of movement planning and organization (e.g., Swinnen et al., 2003).

## BIMANUAL COUPLING IN PRODUCTIVE SYNDROMES

If it is true that coupling effects primarily emerge at the level of movement planning and organization, they should be present whenever an action is planned (even in absence of executed movements and/or feedbacks). Hence, administering bimanual tasks in those pathological conditions in which the movements of one hand cannot be performed can represent an ideal experimental tool to test this hypothesis and to investigate the integrity of motor representations. Indeed, any effect on the “moving” (healthy) hand would be due to the motor constraints imposed by the “non-moving” (impaired) hand. In other words, it is possible to evaluate the effects of the motor programs of one hand without actually requiring its movement. These tasks have been successfully employed to examine productive symptoms (e.g., confabulations), namely “active generation of acts or verbal reports reflecting a distorted mental representation of reality” (Bottini et al., 2009). In these cases, bimanual tasks have revealed whether (or not) productive behaviors are deeply embedded in the patients’ sensory-motor system rather than being a mere verbal confabulation.

### ANOSOGNOSIA FOR HEMIPLEGIA

In AHP, patients show a paresis of the left (contralesional) side of the body following a right hemisphere lesion. Nevertheless, they obstinately deny their motor deficit and, when asked to move their paralyzed limb, they pretend to have performed the requested action (see Pia et al., 2004; Orfei et al., 2007; Bottini et al., 2010; Jenkinson and Fotopoulou, 2010 for reviews). This ‘denial’ may be due to lesions mainly involving pre-motor and insular areas, thought to provide the neural basis of a complex circuit related to motor monitoring (Berti et al., 2005; Karnath et al., 2005; Fotopoulou et al., 2010; Vocat et al., 2010; Moro et al., 2011; Garbarini et al., 2012). This monitoring impairment, in turn, would prevent the patient from distinguishing between movement and no-movement states. However, in AHP there is also a “positive” component, namely the (non-veridical) feeling of having moved. It has been proposed that such productive behavior might arise from a spared activity of the brain structures (such as the SMA, pre-SMA, and CMA) subserving the frontal component of the intention-programming system (Berti and Pia, 2006; Berti et al., 2007; Spinazzola et al., 2008; Garbarini et al., 2012). In other words, anosognosics would still be able to program movements and form predictions, which would lead to the false experience of having moved.

Does this productive behavior reflect the functioning of the same mechanisms that govern normal motor performance? Recently, it has been demonstrated that the motor programs directed to the affected limbs are comparable to those controlling the unaffected limbs (Garbarini et al., 2012; Pia et al., 2013b). Garbarini et al. (2012) employed one of the above-mentioned bimanual paradigms (i.e., circles-lines drawing task; Franz and Ramachandran, 1998) to examine whether in AHP the motor programs directed to the affected limbs are intact. In normal subjects, spatial coupling effects occur when the hand drawing lines produces an “oval” trajectory due to the interference from the other hand drawing circles. The authors found that when anosognosic patients were asked to simultaneously draw lines with their unaffected hand and circles with their paralyzed hand, the trajectories drawn by the intact hand were influenced by the intended but not executed movement of the paralyzed hand and tended to assume an oval shape (spatial coupling effect).

As we mentioned above, however, coupling effects can be found also within the temporal domain. Pia and colleagues (Pia et al., 2013b) examined whether anosognosics show also the same temporal constraints known to exist during bimanual movements in healthy subjects. In these paradigms, when people simultaneously reach for two targets differing in the difficulty of the motor act needed to reach them, the motor programs of one hand interfere with movement execution of the other hand. More precisely, movement time of the hand directed to an easy target (near and large) – while the other hand is aiming to a difficult target (far and small) – is slower if compared to a unimanual movement condition (Kelso et al., 1979). The authors found that, similarly to healthy subjects, anosognosics showed a coupling effect. In bimanual asymmetric conditions, when the non-paralyzed hand aimed at the easy target and the paralyzed hand at the difficult target movement time of the non-paralyzed was slowed down by the “pretended” movement of the paralyzed hand. It is important to note that, in patients without anosognosia neither spatial nor temporal coupling effects were found.

Both above-mentioned studies suggest that, in anosognosic patients, the illusory movements of the paralyzed hand impose to the non-paralyzed hand the same motor constraints that are observed during the actual movements. Rather than being merely a confabulation, anosognosia for the plegic hand can produce measurable constraints on movement execution of the intact hand. It is worth noticing that not all anosognosic patients experience the illusory movements (Feinberg, 2007) that, in our view, are crucial in order to trigger the coupling effects. In future studies, this point could be clarified comparing, during bimanual tasks, the performance of anosognosic patients with and without illusory movement experience.

In sum, these findings clearly show that motor awareness can arise even in absence of movement execution, solely based on normal intentional processes. It is worth noticing that these findings are consistent with previous behavioral (Swinnen et al., 2003) and neuroimaging (Garbarini et al., in press) studies on healthy subjects showing that coupling relies on central operations (i.e., activation of intention/programming system), rather than on online incoming information from the periphery. They are also consistent with the findings of Franz and Ramachandran (1998) who studied amputee patients with a vivid subjective experience of moving their “phantom” limb, describing a bimanual coupling effect similar to that observed in anosognosic patients. A crucial difference between amputees and patients with anosognosia is that the latter’s brain damage prevents them from realizing that their subjective experience is non-veridical. On the contrary, although some amputees can intentionally manipulate their phantom, absence of brain damage allows awareness of the absence of actual movements.

### DELUSION OF OWNERSHIP

In right brain-damaged patients, a body awareness syndrome, known as somatoparaphrenia, can sometimes be observed. Patients may feel a sense of strangeness towards their contralesional limbs that may be acknowledged as separated from their own body. The more frequent manifestation of this disorder is a sense of disownership, namely the delusional belief that the contralesional limbs do not belong to one’s own body but to another person’s (Vallar and Ronchi, 2009; Jenkinson et al., 2013). From an anatomical point of view, white matter and some subcortical structures (i.e., thalamus, basal ganglia, and amygdala) seem to have a key role for the emergence of this deficit (Gandola et al., 2012). Recently, it has been reported the existence of an opposite condition, that is that of patients who misidentify other people’s limbs as their own (Garbarini et al., in press; Pia et al., 2013a). These patients, while not explicitly denying that their contralesional (left) arm belongs to themselves (as in the somatoparaphrenic delusion of disownership), claim that the examiner’s left hand is their own whenever it is positioned, in egocentric coordinates, next to their left arm. These patients treat and care for the experimenter’s left arm as if it was their own, showing a consistent embodiment of the alien hand in their own body schema. It is worth noticing that such a delusion of ownership, although resembling the “rubber-hand-illusion” (Botvinick and Cohen, 1998), is spontaneous and stable, not transiently induced by an experimental manipulation. Consistently with previous data on the neural correlates of the delusion of disownership (Gandola et al., 2012), preliminary data showed that delusion of ownership might be related also to damage to basal ganglia and dorsolateral prefrontal cortex, with a sparing of mesial prefrontal areas, such as SMA, pre-SMA, and CMA (Garbarini et al., in press; Pia et al., 2013a).

Given the tight link between body and motor representations (Gallese and Sinigaglia, 2010), one might ask whether an altered sense of body-ownership also affects patients’ motor awareness and sense of agency. One possibility is that, once an alien hand is embodied into the patient’s body schema, its representation can affect motor production and motor control as if it actually belonged to the patients. Garbarini et al. (in press) asked these patients to execute a modified version of the bimanual circles-lines task (Franz et al., 1991). Patients had to draw lines with their intact hand while watching an “alien” (embodied) hand performing circles, either in an egocentric position (i.e., congruent with the position of the patients’ left hand) or in an allocentric non-congruent position (i.e., positioned in front of the patient). The crucial aspect of this experiment was that in the congruent condition, these patients misidentified the alien hand as their own, while in the non-congruent condition they recognized the alien hand as belonging to the experimenter. If the delusion of ownership arises from a pathological embodiment that automatically triggers the intention-programming processes for one’s own hand, when the alien hand draws circles in the egocentric condition, the lines drawn by the patient’s intact hand should become ovalized (coupling effect), as in normal individuals actually performing the bimanual task. Indeed, these patients clearly showed the effect in the alien congruent (egocentric) condition (in which patients claimed that they performed circles with their left hand). It is important to note that, in the same condition, neither healthy controls nor hemiplegic patients without embodiment showed any coupling effect, suggesting that simply looking at a hand drawing circles is not sufficient to induce a coupling effect. Consistently, only patients with the delusion claimed that they performed circles with their left hand, suggesting that the altered sense of ownership influenced the sense of agency. When they were asked to say how was it possible that they performed circles with their paralyzed hand, they produced confabulations: “I performed the movement with the power of my thought”; “Maybe the examiner helped me in performing circles”; “Wonderful! I got well!”

These findings demonstrate the existence of a deeply altered sense of body ownership affecting both motor awareness (patients, normally aware of their motor impairment, were convinced that their left hand was moving) and sense of agency (patients ascribed the alien movements to themselves). This, in turn, directly influences action execution (patients showed an interference/coupling effect very similar to those found in healthy subjects actually performing bimanual circles-lines task). It is important to emphasize that these patients, although comparable to anosognosic patients with respect to the presence of hemiplegia, are, however, behaviorally different. Indeed, in an ecological condition they acknowledge their motor deficit, therefore showing normal motor awareness and motor monitoring. However, the presence of an alien hand positioned next to their contralesional hand dramatically affects their motor consciousness. The circles-lines task employed in this experiment showed that the pathological embodiment of alien body parts could not only alter one’s own body schema, but also action execution.

## CONCLUSION AND FUTURE DIRECTIONS

In the present review, we report the findings of recent neuropsychological studies employing the bimanual coupling effect in order to analyze productive motor behaviors in brain-damaged patients. These studies unveiled information about the internal motor representation of actions of one arm independently from its movements. Additionally, they opened a window onto the mechanisms subserving bimanual coordination in the intact brain, supporting the view that internal mechanisms might be sufficient to produce bimanual coordination effects.

Although a detailed discussion of the neural correlates of the above described productive behaviors is beyond the scope of the present review, it is interesting to note that, as mentioned above, patients with both AHP and delusion of ownership are supposed to have no lesions in the right medial motor areas – the SMA and the underlying CMA – showing a prominent role in mediating the coupling effect during bimanual non-congruent movements. This suggests that these patients were potentially able to plan (not to perform, due to the paralysis) bimanual non-congruent movements giving rise to coupling effects. Future neuroimaging studies might extend the use of bimanual paradigms to investigate the neural correlates of the “illusion of movement,” described in pathological situations such as the phantom limb, AHP or delusion of ownership in which previous behavioral studies (Franz and Ramachandran, 1998; Garbarini et al., 2012; Garbarini et al., in press) suggested the presence of bimanual coupling effects even in the absence of actual movement execution. Furthermore, future research could investigate the presence and the structure of internal motor representation of actions in other neurological conditions in which the movements of one hand is absent or partially impaired [e.g., apraxia (Vanbellingen and Bohlhalter, 2011), such as motor neglect (Laplane and Degos, 1983), or ataxia (Bastian, 1997)], or in psychiatric disorders (e.g., schizophrenia) that manifest a delusion of awareness of one’s own action as well as of recognition of actions performed by others (i.e., Daprati et al., 1997).

## Conflict of Interest Statement

The authors declare that the research was conducted in the absence of any commercial or financial relationships that could be construed as a potential conflict of interest.
